# Peripherin, A New Promising Biomarker in Neurological Disorders

**DOI:** 10.1111/ejn.70030

**Published:** 2025-02-24

**Authors:** Carlo Manco, Delia Righi, Guido Primiano, Angela Romano, Marco Luigetti, Luca Leonardi, Nicola De Stefano, Domenico Plantone

**Affiliations:** ^1^ Centre of Precision and Translational Medicine Department of Medicine, Surgery, and Neuroscience University of Siena Siena Italy; ^2^ Department of Neurosciences, Sensory Organs and Thorax Agostino Gemelli University Hospital Foundation IRCCS Rome Italy; ^3^ Department of Neurosciences Catholic University of the Sacred Heart Rome Italy; ^4^ Neuromuscular and Rare Disease Centre Neurology Unit Sant'Andrea Hospital Rome Italy

**Keywords:** amyotrophic lateral sclerosis, biomarker, neurofilaments, Peripherin, polyneuropathy

## Abstract

Peripherin is a class III intermediate filament protein that has recently gained attention as a potential biomarker for axonal damage in the peripheral nervous system. This review examines peripherin gene expression, protein structure, and its functions in both healthy and diseased states. Peripherin is predominantly expressed in the peripheral nervous system, especially in motor and sensory neurons, and plays a critical role in neurite growth, stability, and axonal transport during myelination. Its expression is regulated by various cytokines and undergoes several post‐transcriptional modifications. Peripherin interacts with multiple proteins, including neurofilaments and kinases, influencing cytoskeletal dynamics and neuronal functions. The review also explores peripherin involvement in several neurological disorders, such as Amyotrophic Lateral Sclerosis, where its abnormal expression and aggregation contribute to disease pathology. Additionally, peripherin has been linked to polyneuropathies, traumatic axonal injury, and diabetic neuropathy, suggesting its broader relevance as a biomarker in these conditions. The potential of peripherin as a biomarker is further supported by recent studies using ultrasensitive detection methods, which have identified elevated peripherin levels in the serum of patients with neurological diseases. Despite the promising findings, the application of peripherin as a biomarker in clinical settings remains limited, primarily due to challenges in its detection and the need for further validation in diverse patient populations. Future research directions include the development of more sensitive assays and the exploration of peripherin's role in non‐neuronal tissues, which may expand its diagnostic and therapeutic potential.

## Graphical abstract

Peripherin is a class III intermediate filament protein, primarily found in motor and sensory neurons of the peripheral nervous system. It plays a key role in neurite growth, axonal transport, and myelination. Its expression is regulated by cytokines and undergoes post‐transcriptional modifications, interacting with neurofilaments. Abnormal peripherin expression is linked to neurological disorders, including ALS and neuropathy. Peripherin shows potential as a biomarker for axonal damage. However, clinical applications are limited by detection challenges, requiring further research and development.

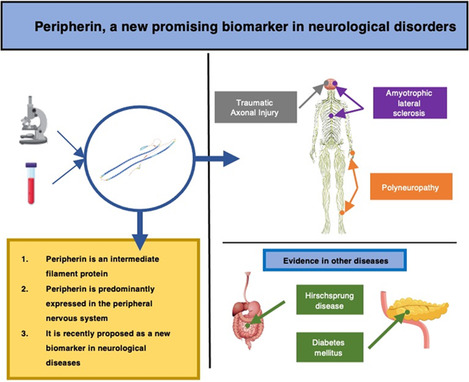

**Abbreviations**
CNS: Central nervous system
NGF: Neuronal Growth Factor
FGF: Fibroblast growth factor
RAB7A: Ras‐related protein Rab‐7a
SNAP‐25: Synaptosome associated protein 25
SIP30: Interacting protein 30,
NfL: Neurofilament light chain
sAPP: Amyloid‐β precursor protein
PKCα: Protein kinase C alpha
PKCε: Protein kinase C epsilon
ALS: Amyotrophic lateral sclerosis
GBS: Guillain Barrè syndrome
CIDP: Chronic inflammatory demyelinating polyradiculopathy
TAI: Traumatic axonal injury
TBI: Severe traumatic brain injury
DAI: Diffuse axonal injury
DM‐I: Diabetes mellitus type I
HSCR: Hirschsprung disease
EV‐A71: Enterovirus‐A71
MND: Motor neuron diseases
MS: Multiple sclerosis
RT‐qPCR: Quantitative real‐time PCR
SMA: spinal muscular atrophy
PN: peripheral neuropathy
αS: α‐synuclein

## 1 Introduction

Peripherin is an intermediate filament protein of class III that has recently garnered attention as a potential biomarker for axonal damage in the peripheral nervous system (Romano, Del Fiore, and Bucci 2022; Thompson and Ziff 1989). This interest arises from its distinct expression pattern, which is predominantly found in the spinal cord ventral horn, alpha motor neurons, and primary afferent sensory neurons in the dorsal columns (Leonard et al. 1988; Parysek and Goldman 1988; Romano, Del Fiore, and Bucci 2022). Although the precise functions of peripherin are not fully understood, it is believed to play a crucial role in neurite growth, stability, and axonal transport during myelination. The aim of this systematic literature review is to consolidate current knowledge on peripherin by emphasizing its unique characteristics, highlighting its potential role as a biomarker in neurological diseases, and exploring its possible applications in other pathological conditions.

## 2 Method

To select the relevant literature for this systematic review the authors conducted searches on Pubmed and Embase. This systematic review was conducted following PRISMA (Preferred Reporting Items for Systematic Reviews and Meta‐Analyses) guidelines. Different filters were applied according to the sections of the review. All authors worked independently and all used Mendeley as reference software for data management. Each study was included only if it had not already been included by another author on the shared platform. For paragraph 3, no filter was used; Romano, Del Fiore, and Bucci 2022’s (Romano, Del Fiore, and Bucci 2022) work was used as the main reference work, the search was based on the keywords “peripherin” AND “gene”, “peripherin” AND “protein expression”, “peripherin” AND “functions”, while for paragraph 3.1 all articles with the keywords “peripherin” AND “interaction” were included without temporal and species filter; articles related to peripherin‐2 (PRPH‐2) were excluded (Reason 1). For paragraphs 4.1 to 4.5, only articles published from 2000 to 2024 involving human species and with the following keywords: “peripherin” AND “assay”, peripherin” AND “enzyme‐linked immunoassay”, “peripherin” AND “analysis”, “peripherin” AND “neuropathy”, “peripherin” AND “neurological disease”, “peripherin” AND “amyotrophic lateral sclerosis”, “peripherin” AND “polyneuropathy”, “peripherin” AND “axonal injury”, “peripherin” AND “diabetes mellitus”, “peripherin” AND “autoimmune disease”, “peripherin” AND “enteric disease”, “peripherin AND “Hirschsprung disease”, “fluid biomarker” AND “Hirschsprung disease”, “serum biomarker” AND “Hirschsprung disease”, “biomarker” AND “Hirschsprung disease” were selected. For paragraph 4.6 only articles published since 2000 and based on the keywords: “peripherin” AND “infection”, “peripherin” AND “synuclein”, “peripherin” AND “neuroblastoma” were included. For paragraphs 5 to 5.4, the same keywords of paragraphs 4.1 to 4.5 and only articles published since 2000 were used. Articles that were deemed important were entered independently of the time filter.

**FIGURE 1:**
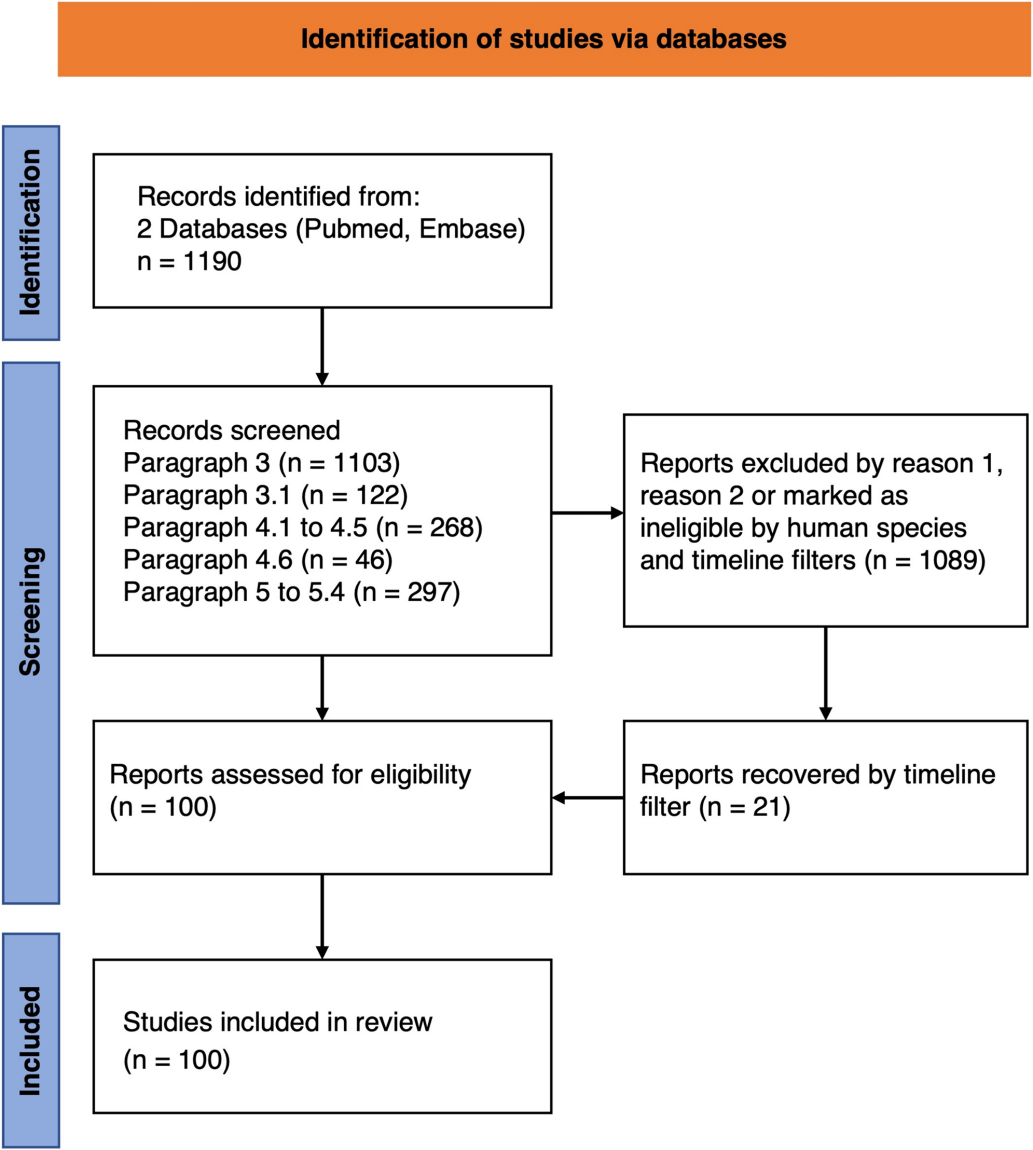
Representation of the identification process (from the Pubmed and Embase databases), screening, and inclusion of the mentioned articles. The literature selection for this systematic review was carried out following the PRISMA guidelines. During the screening phase, articles referring to peripherin‐2 were excluded (Reason 1), as well as those that did not address at least one of the three main objectives of each paragraph (genetic aspects, neuropathological aspects, or potential as a biomarker of peripherin. Reason 2).

For each paragraph, the outcomes aimed to describe: the (I) genetic aspects, (II) neuropathological, and (III) the potential as biomarker of peripherin. Only articles that addressed at least one of the three objectives (genetic aspects, neuropathological aspects, or potential as a biomarker) were selected in the screening phase (Reason 2). The search and selection process are shown in Figure 1 while in Table 1 the keywords for each paragraph are reported.

**TABLE 1:** Table shows the main paragraphs and the keywords for each of them.

Paragraph Title	Keywords
3. Peripherin: Gene, protein expression and its functions	Gene, protein expression and post‐transcriptional modifications, myelination
3.1 Peripherin interaction in neuronal cells	Protein interactions, neurofilaments, amyloid‐β precursor protein, protein kinase
4.1 Amyotrophic lateral sclerosis (ALS)	Genetic variants, neurotoxic effects, biomarkers
4.2 Polyneuropathy	Genetic variants, loss of function
4.3 Traumatic axonal injury (TAI)	Axonal damage, protein expression
4.4 Diabetes mellitus and Neuroendocrine autoimmune manifestation	Autoimmune endocrine disease, neuropathy, pancreatic cancer
4.5 Enteric nervous system disease	Hirschsprung disease, Immunohistochemistry
4.6 Initial evidence in other diseases	Enterovirus‐A71, amyloidogenic α‐synuclein
5. Peripherin as a biomarker: Current applications and future directions	Fluid biomarker, immunohistochemistry
5.1 ALS/MND	CSF and serum biomarker, ELISA
5.2 Traumatic axonal injury	Plasma biomarker, immunohistochemistry, ELISA, and morphological analysis
5.3 Peripherin as a possible biomarker in Guillain Barrè Syndrome	Serum biomarker, neurofilament light chain, Simoa method
5.4 Hirschsprung disease	Serum microRNA, immunohistochemical biomarker

## 3 Peripherin: Gene, Protein Expression and Its Functions

Peripherin, a 58 kDa insoluble protein, is classified as a class III intermediate filament protein based on its genetic sequence and structure, first discovered in 1983 (Portier, de Néchaud, and Gros 1983; Portier, Brachet et al. 1983). Biochemical studies have revealed that its expression is predominantly in the peripheral nervous system, hence its name (Portier, de Néchaud, and Gros 1983; Zhao and Liem 2016). Genetically, it is composed of nine exons and eight introns and is highly conserved between humans and mice (Foley, Ley, and Parysek 1994). Conserved domains are located in introns 1 and 2 and in the 5′ flanking region, which includes PER1, PER2, PER3, NGFNRE, AP‐2, Hox A5 consensus sequence and one potential binding site of Heat Shock Protein (Foley, Ley, and Parysek 1994; Romano, Del Fiore, and Bucci 2022). It has been suggested that the second ATG site represents the preferred translation initiation codon for peripherin gene (Ho et al. 1995).

Peripherin exists in several isoforms, generated by the differential splicing of the dominant 58 kDa form (McLean et al. 2008). Each isoform is named based on its molecular weight. These isoforms include Per‐28 resulting from the retention of introns 3 and 4 and a premature stop codon in intron 3 (Xiao et al. 2008), Per‐32 resulting from the retention of intron 4 and a premature stop codon, Per‐45 initiated by an in‐frame downstream initiation codon (McLean et al. 2008), and alternative splicing in exon 9 producing Per‐56 (Landon et al. 1989). Additionally, a 32‐amino acid insertion due to the retention of intron 4 results in Per‐61 (Robertson et al. 2003). Per‐61 is expressed only in mice, it is not expressed in humans following genetic splicing mechanisms; in fact, intron 4, required for the formation of Per‐61, is retained for the formation of Per‐32 preventing its expression (Romano, Del Fiore, and Bucci 2022; Xiao et al. 2008) (Table 2).

**TABLE 2:** Isoforms of peripherin. Isoforms of the peripherin protein in mice and human, their alternative splicing and function known.

Isoform	Species expression	Alternative splicing and function	References
Per‐28	Human	Retention of the intron 3 and 4 plus and a premature stop codon in intron 3 This isoform is expressed at low stoichiometric and when upregulated cause peripherin aggregation Mild neurotoxicity form	(Xiao et al. 2008)
Per‐32	Human	By retention intron 4 and premature stop codon	(Romano, Del Fiore, and Bucci 2022)
Per‐45	Mice and human	In‐frame downstream initiation codon Required for organization of a normal filament network, co‐assemble with Per58	(McLean et al. 2008)
Per‐56	Mice and human	Splice site in exon 9 Isoform capable to stable a normal filamentous network alone	(Landon et al. 1989)
Per‐58	Mice and human	Predominant form Isoform capable to stable a normal filamentous network alone	(McLean et al. 2008)
Per‐61	Mice	32 amino‐acid insertion by retention of intron 4 Cannot form a filament network Neurotoxic form	(Robertson et al. 2003; Romano, Del Fiore, and Bucci 2022)

Abbreviation: Per, peripherin.

In mice, peripherin expression begins later (at stage 34‐somite) than neurofilament light chain (NF‐L), coinciding with the migration of neural cells reaching their destinations and terminally differentiating into various types of neurons. Peripherin expression is localized in the ventral horn motor neurons of the spinal cord and is distributed in the sciatic nerve, autonomic ganglionic and preganglionic neurons, and sensory neurons (Escurat et al. 1990; Yuan et al. 2012). Expression in the central nervous system (CNS) is predominantly found in the brain stem, optic tract, and internal capsule (Fang et al. 2023). Specifically, it was present in the nerve fibres and nuclei associated with cranial nerves V to XII and is prominent in the cell bodies and axons of the mesencephalic trigeminal nucleus, the pars compacta region of the nucleus ambiguus, as well as in fibres that comprise the solitary tract, the descending spinal trigeminal tract, and the trigeminal and facial nerves. Additionally, peripherin‐positive fibres were observed in the inferior cerebellar peduncle and the folia of the intermediate zone of the cerebellum (Barclay et al. 2007). Peripherin positivity has been observed in the corpus callosum in murine models and patients with axonal injury (Fang et al. 2023). *PRPH* expression has been demonstrated also in pancreatic islet beta‐cells (Boitard et al. 1992) and interestingly, autoreactive B cells characterized by a specific immune response to peripherin have been demonstrated to have a significant role in the non‐obese diabetic mouse model, representing a heterogeneous population proliferating as diabetes develops (Song et al. 2009).

Various cytokines regulate *PRPH* expression and post‐transcriptional modifications, such as Neuronal Growth Factor (NGF) (Aletta, Shelanski, and Greene 1989), IL‐6 (Sterneck, Kaplan, and Johnson 1996), and fibroblast growth factor (FGF) (Choi et al. 2001). NGF has been shown to increase the phosphorylated form of peripherin, impacting its solubility and dynamics, and consequently, its role in network organization (Aletta, Shelanski, and Greene 1989). Other post‐transcriptional modifications were highlighted as acetylation, nitration and methylation but the significance is not entirely understood (Cappelletti et al. 2006; Petzold 2022; Tedeschi et al. 2007).

Evidence in the literature supports the role of peripherin in neurite growth, potentially aiding in the recognition of axonal pathways after the migration phase of neural crest cells and following neuronal injury (Beaulieu, Kriz, and Julien 2002; Gorham et al. 1990; Oblinger, Wong, and Parysek 1989; Troy, Brown et al. 1990; Troy, Muma et al. 1990). It also plays a role in neurite stability, particularly in long myelinated neurons (Helfand et al. 2003; Larivière et al. 2002). Moreover, peripherin interact with proteins such as Ras‐related protein Rab‐7a (RAB7A ‐ a protein involved in the late endocytic pathway) (Cogli et al. 2013) or Adaptor protein complex 3 (AP‐3) (Styers et al. 2004), which are engaged in endosome and lysosome transport, and synaptosome associated protein 25 (SNAP‐25) interacting protein 30 (SIP30), which is involved in SNAP receptor‐dependent exocytosis (Gentili et al. 2014). These interactions with proteins essential for vesicular trafficking and axonal transport suggest that peripherin may play a direct role in these cellular functions. In addition, peripherin expression appears to modulate the transport of organelles, such as mitochondria and lysosomes. A time‐lapse microscopy study on cultured neurons derived from primary dorsal root ganglia of mice (i) lacking neurofilament light chain, (ii) overexpressing peripherin, or (iii) double transgenic (combining both conditions) demonstrated that retrograde mitochondrial transport was increased in double transgenic neurons, whereas anterograde transport was enhanced in cultures with peripherin overexpression and in those lacking neurofilament light chain. These findings highlight a role for peripherin in regulating the transport of these organelles (Perrot and Julien 2009).

### 3.1 Peripherin Interactions in Neuronal Cells

Peripherin has been shown to interact with numerous molecules, in particular with neurofilament subunits (Parysek et al. 1991) and together interact with microtubules through kinesin and dynein favouring their axonal transport (Shah et al. 2000; Yabe, Pimenta, and Shea 1999). An interesting relationship was described with soluble amyloid‐β precursor protein (sAPP). Following APP cleavage, sAPP associates with perinuclear peripherin (Muresan, Villegas, and Muresan 2014). Furthermore, sAPP, similar to other proteins implicated in neurodegenerative processes, such as TDP‐43, FUS, and SOD1, is transported to neurite terminals through a mechanism mediated by peripherin (Muresan and Muresan 2016).

It has been shown that peripherin is a substrate for several protein kinases. For example, the serine/threonine kinase Akt, also known as protein kinase B, interacts with the head domain of peripherin, and the induction of its phosphorylated form promotes nerve regeneration (Konishi et al. 2007; Read and Gorman 2009). Similarly, peripherin is a substrate of protein kinase C alpha (PKCα) and a target of ATP‐dependent PKCα signalling. Proper control of PKCα is crucial for the assembly of peripherin filaments, as its downregulation dramatically impacts peripherin organization in NGF and ATP‐differentiated PC12 cell cultures (Marín‐Vicente et al. 2011).

A negative effect on peripherin, leading to its aggregation and subsequent cell death, is caused by the interaction with the C1b domain of protein kinase C epsilon (PKCε) in neuroblastoma cells. This finding highlights the potential therapeutic opportunities of targeting specific protein interaction sites (Sunesson, Hellman, and Larsson 2008). Additionally, the function of peripherin is regulated by a ubiquitous GTPase, RAB7A, which, when overexpressed or silenced, modifies the ratio between soluble and insoluble peripherin (which will be discussed in more detail in paragraph 4.2 (Cogli et al. 2013).

It has been documented that peripherin is upregulated following an axonal injury (Hol and Capetanaki 2017; Wong and Oblinger 1990). During regeneration (Portier et al. 1993), peripherin can self‐assemble to establish an intermediate filament network or interact and assemble with other neurofilaments, including syncoilin, an atypical type III intermediate filament protein expressed in the central and peripheral nervous systems (isoforms Sync1 and Sync2) (Clarke et al. 2010) and NfL (Beaulieu, Robertson, and Julien 1999). Finally, a yeast two‐hybrid screen on a mouse brain cDNA library using an assembly‐incompetent peripherin isoform, a Per‐61 as bait, identified interactors involved in various cellular processes. These include vesicular trafficking (SNAP25 interacting protein 30, vacuolar proton pump subunit D, etc.), signal transduction (transforming growth factor beta regulator 1, hepatocyte growth factor regulated tyrosine kinase substrate), DNA/RNA processing (zinc finger protein 219, etc.), protein folding (E3 ubiquitine protein ligase, etc.), and mitochondrial metabolism (mitochondrial ribosomal protein L38, Mitofusin 1, etc.) demonstrating the involvement of peripherin in multiple cellular functions essential for maintaining cell homeostasis (Gentili et al. 2014). Therefore, peripherin is essential for various cellular functions beyond its primary structural role.

## 4 Peripherin in Human Diseases

The discovery of this new class III intermediate neurofilament has opened a new field of research, highlighting its involvement in numerous neurological diseases and beyond.

### 4.1 Amyotrophic Lateral Sclerosis

One of the initial and pivotal implications of peripherin has been its association with amyotrophic lateral sclerosis (ALS) (Xiao, McLean, and Robertson 2006). Interestingly in both sporadic and familial forms of ALS hyperphosphorylated peripherin and neurofilaments have been found in motoneurons, axonal spheroids, and perikaryal deposits (Manetto et al. 1988). Evidence in the literature suggests that certain splicing isoforms of peripherin, such as the 61 kDa (Per‐61) (Robertson et al. 2003) and 28 kDa (Per‐28) forms (Xiao et al. 2008) when expressed or overexpressed, exhibit neurotoxic effects and may lead to protein aggregation. This imbalance in peripherin isoforms can result in protein accumulation, blockage of cellular transport (Millecamps et al. 2006), and cellular metabolic alterations, a specific condition for ALS (Beaulieu and Julien 2003; McLean et al. 2008). Dysregulation of microRNAs, such as microRNA‐105 and ‐9, which are important regulators of peripherin expression, may contribute to instability in peripherin mRNA levels, as demonstrated by Hawley et al. in 2019 (Hawley, Campos‐Melo, and Strong 2019). The discovery of peripherin in ubiquitinated inclusions and the identification of genetic variants of the peripherin gene, particularly the insertion *PRPH* (IVS8)(−36insA), deletion *PRPH* (228delC), and missense variations c.421G > T (p.D141Y) and c.398 G > C (p.R133P) in ALS patients, which disrupt the assembly of the neurofilament network in transfected cells, further supporting its role in pathogenesis of this neurodegenerative disorder (Corrado et al. 2011; Gros‐Louis et al. 2004; Leung et al. 2004) (Table 3). Additionally, immunostaining studies have shown that peripherin is one of the most common intermediate neurofilaments found in motor neuron spheroids in ALS patients (Corbo and Hays 1992). The presence of peripherin in Bunina bodies and Lewy body‐like inclusions, which are protein aggregates found in the cell body of motor neurons of subjects with this specific neurological disease, has also been demonstrated through immunohistochemistry evaluation (He and Hays 2004; Mizuno et al. 2011), suggesting a potential role in their formation (Miki et al. 2018).

**TABLE 3:** Disease and neurological condition associated with peripherin gene variants.

Variants	Disease	References
PRPH IVS8‐3einsA	ALS	(Gros‐Louis et al. 2004)
PRPH 228delC (p.Arg77fs)	ALS	(Gros‐Louis et al. 2004)
PRPH c.421G > T (p.D141Y)	ALS	(Leung et al. 2004)
PRPH c.398G > C (p.R133P)	ALS	(Corrado et al. 2011)
PRPH c.966 + 1G > A	Reduced nerve conduction amplitude	(Bjornsdottir et al. 2019)

Abbreviations: ALS, amyotrophic lateral sclerosis; PRPH, peripherin.

Peripherin is involved in TDP‐43 aggregates transport towards the axonal terminals. Knocking down peripherin disrupts the neurofilament network and reduces TDP‐43 accumulation (Muresan and Muresan 2016). The significance of serum peripherin as a clinical biomarker in motor neuron disease will be reviewed later.

### 4.2 Polyneuropathy

Alterations in proteins interacting with peripherin can cause polyneuropathy. For example, the genetic variant c.377A > G (p.K126R) appears to expand the phenotypic spectrum of CMT2B, which is primarily considered a sensory neuropathy, towards symptoms with motor predominance. The c.377A > G (p.K126R) genetic variants has been shown to alter the biochemical properties of RAB7 GTPase, reducing GTPase activity. This reduction has a negative impact on neurite outgrowth and axonal regeneration. Additionally, the variant increases interaction with peripherin, potentially interfering with its assembly and impairing axonal regeneration. Finally, this mutation appears to inhibit EGFR degradation, leading to its accumulation and a potential increase in signalling that could further interfere with nerve regeneration (Saveri et al. 2020). Similarly, deficits in protein interactions appear to underlie Giant Axonal Neuropathy, an early‐onset neuropathy characterized by intermediate filament aggregates. Pathogenic variants in the *GAN* gene, which encodes Gigaxonin (an E3 ligase adaptor) lead to the accumulation of intermediate filament aggregates. A study on human fibroblasts has elucidated Gigaxonin's role in peripherin and vimentin (another intermediate filament) degradation and restoration of the missing gene can clear neuronal cells of intermediate filament accumulations, restoring cytoskeletal homeostasis (Mahammad et al. 2013; Mussche et al. 2013).

Furthermore, a significant study involving 7045 Icelanders identified a low‐frequency variant of the *PRPH* gene (c.966 + 1G > A) associated with reduced nerve conduction amplitude, though not velocity (Table 3). This variant leads to peripherin loss of function and, when overexpressed, alters filament structure and forms protein inclusions, predisposing individuals to mild axonal polyneuropathy with early and sensory‐negative symptoms (Bjornsdottir et al. 2019).

### 4.3 Traumatic Axonal Injury (TAI)

Traumatic axonal injury (TAI) is a condition characterized by widespread damage to the axons of cerebral white matter, often affecting the corpus callosum and brainstem, typically occurring following severe traumatic brain injury (TBI). Mechanisms underlying axonal damage appear to involve cytoskeletal disruption, accompanied by reactive alterations in the expression of structural proteins (Johnson, Stewart, and Smith 2013; Smith, Meaney, and Shull 2003). In a study using a mouse model of diffuse axonal injury (DAI), combined with bioinformatics analysis of expressed proteins, Liang et al. (2019) identified significant upregulation of peripherin and calsenilin during DAI particularly post‐lesion, suggesting their potential as biomarkers (Liang et al. 2019). Subsequent investigations by the same authors, employing murine models of TBI and human brain tissue post‐TBI, revealed elevated peripherin levels in CSF and plasma, along with co‐localization of peripherin with other biomarkers such as NfL (in the corpus callosum) and amyloid precursor protein (in the brainstem), indicating peripherin involvement in mechanisms of neuronal fibre structure restoration and maintenance following TBI. Histological analysis of human tissue samples further confirmed peripherin's diagnostic utility in identifying damaged axons (Fang et al. 2023).

### 4.4 Diabetes Mellitus and Neuroendocrine Autoimmune Manifestation

Several scientific papers have emphasized the connection between autoimmune endocrine diseases and neurological manifestations, specifically neuropathy. A large study, performed by a blind screening of 160,000 sera for neurological autoimmunity markers, identified 26 patients with IgG selectively binding peripherin and a high rate of dysautonomia and moderate neuropathy, 35% had endocrinopathy (between diabetes mellitus 1, thyroiditis, and premature ovarian dysfunction). Among patients with small fibre/autonomic neuropathies (with or without endocrinopathy) seropositivity for peripherin IgG was found in 33% (Chamberlain et al. 2010). A subsequent study has confirmed that phosphorylated peripherin (in dimeric conformation) may be a candidate antigen for diabetes mellitus type I (DM‐I). Doran and colleagues found anti‐peripherin phosphorylated antibodies in 67% of serum donors with DM‐I (Doran et al. 2016).

Peripherin gene becomes overexpressed also in pancreatic cancer and a high association has been demonstrated with diabetes mellitus, raising therefore the question of whether an autoimmune mechanism can be the basis of the development of DM related to pancreatic cancer (Song et al. 2009). However, it remains unclear if autoimmunity against peripherin is the primary autoimmune target leading to endocrine autoimmune disease. Nevertheless, peripherin in diabetes mellitus seems to play a role not only as an immune target but also as a molecule for neuronal wellness. A higher rate of gastrointestinal symptoms (constipation and diarrhoea) in diabetic patients has been associated with a significant reduction in the size of the colonic ganglia, loss of peripherin, and increased neuronal apoptosis (Chandrasekharan et al. 2011). In this direction, the role of peripherin in diabetic patients remains of extreme interest also for its prognostic and clinical implications.

### 4.5 Enteric Nervous System Disease

Peripherin has emerged as a promising biomarker for investigating enteric nervous system malformations. It has demonstrated efficacy as an immunohistochemical biomarker of submucosal ganglion cells (Matsuda et al. 2006; Szabolcs et al. 1996), particularly useful in the exclusion of Hirschsprung disease (HSCR) by detecting ganglion cells and nerve fibrils more efficiently than biomarkers as MAP‐2 and Calretinine (Chisholm and Longacre 2016). A protocol for the immunohistochemistry evaluation (Galazka et al. 2020) and a diagnostic algorithm incorporating other biomarkers (Holland et al. 2010) have been proposed for the diagnosis of HSCR, offering opportunities for differential diagnosis (Becker and Jensen 2024; Chaffin et al. 2016). Utilizing peripherin as an enteric neuronal marker, differences have been observed between the ganglion cells and nerve fibres of patients with normal internal anal sphincter, a reduced number of nerve fibres of patients with internal anal sphincter achalasia, absence in patients with Hirschsprung disease with a hypertrophic nervous trunk in the letter group (Piotrowska, Solari, and Puri 2003).

### 4.6 Initial Evidence in Other Diseases

Research conducted on infected mouse cell lines (neurons and neuroblastoma) has revealed the involvement of peripherin in Enterovirus‐A71 (EV‐A71) infection. Specifically, it appears to facilitate viral entry and influence viral replication, colocalizing with viral particles in muscles, neuromuscular junctions, and the spinal cord. Furthermore, recent findings have indicated that intracerebral injection of amyloidogenic α‐synuclein (αS) in wild‐type human αS transgenic mice induces αS intracellular inclusions, gliosis, and neuronal damage. Aberrant induction of peripherin expression was observed predominantly at the injection sites associated with neuronal injury (Sacino et al. 2014). This latter evidence is particularly relevant because it shows how neuronal injury may induce peripherin expression (Beaulieu, Kriz, and Julien 2002). Additionally, peripherin demonstrates promise as an immunohistochemistry marker for diagnosing neuroblastoma, with high efficiency in the differential diagnosis of small round cell tumours of childhood (Willoughby et al. 2008).

## 5 Peripherin as a Biomarker: Current Applications and Future Directions

Currently, the use of peripherin measurement in biological fluids is quite limited, and indeed, very few studies have addressed this topic. The main reason why peripherin has not, until very recently, been considered a reliable biomarker is likely because traditional ELISA methods did not allow for adequate quantification of peripherin in most pathologies and in healthy subjects. The most important study exploring the role of peripherin as biomarker in neurological diseases was recently published by Keddie and colleagues (Keddie et al. 2023a). In this study, the authors developed a Simoa immunoassay performed with the homebrew technology by using 8G2 anti‐peripherin as capture antibody (Sigma‐Aldrich) and A‐3 anti‐peripherin mouse (Santa‐Cruz) as detector antibody, applied to a cohort of patients with Guillain‐Barré Syndrome. The homebrew technology is based on the adaptation of the Simoa platform (a technology based on a highly sensitive immunoassay that allows the measurement of biomarkers at the single‐molecule level) for the creation of custom tests, developed internally by laboratories to measure specific biomarkers or analytes not yet available in commercial kits. In this section, we will discuss the very few attempts to use peripherin as biomarker in human biofluids and tissues.

### 5.1 ALS/MND

The first and, to date, the only study that has investigated serum and CSF levels of peripherin in patients with motor neuron diseases (MND) was conducted by Sabbatini and colleagues in 2021 (Sabbatini et al. 2021). The authors performed a retrospective longitudinal study, recruiting 160 subjects evaluated at a single centre from 2016 to 2020, of whom 91 were diagnosed with MND (specifically, 63 with probable or definite ALS, 20 with Spinal Muscular Atrophy type 3, and 8 with Spinal and Bulbar Muscular Atrophy, for whom only serum samples were available for measurement), 26 with dementia, 14 with peripheral neuropathy, and 29 healthy controls who underwent lumbar puncture due to a clinically suspected, but ultimately unconfirmed, neurological disease. The authors measured the levels of NfL and peripherin in CSF and serum samples using ELISA methodology with an analytical sensitivity set <0.156 ng/mL and calibrators 0.312–20 ng/mL. They found median serum peripherin levels of 5.376 ng/mL in ALS, 5.653 ng/mL in spinal muscular atrophy (SMA), and 10.224 ng/mL in spinal and bulbar muscular atrophy, compared to 3.168 ng/mL in healthy controls, 1.793 ng/mL in dementia, and 2.21 ng/mL in peripheral neuropathy (PN). Similarly, NfL values were significantly higher in the ALS group, with a median of 4357.8 pg/mL, compared to 376.6 pg/mL in healthy controls, 168.0 pg/mL in SMA, and 1016.7 pg/mL in PN. There was no significant difference between the ALS and dementia groups (median dementia group 2923.4 pg/mL). The values reported by Sabbatini et al. (2021) were in the ng/mL range and showed a large variability, probably due to the relatively low sensitivity of ELISA compared to newer techniques (e.g. Simoa technology). It is noteworthy that the peripherin levels in CSF were found to be below the detection limit of the assay's calibration curve. The higher concentration of peripherin in serum compared to CSF was attributed to the origin of this biomarker from the cytoskeleton of neurons within the peripheral nervous system (Yuan et al. 2012).

Serum peripherin concentrations were elevated in all patients with MND compared to healthy controls, with particularly higher levels observed in patients with Spinal and Bulbar Muscular Atrophy, likely due to the concomitant sensory axonal neuropathy commonly found in this patient group (Hama et al. 2012). In contrast, CSF NfL levels were elevated in patients with ALS and dementia, corroborating the role of this biomarker in these neurodegenerative diseases (Narayanan et al. 2021; Khalil et al. 2024, 2018). No correlation was found between serum peripherin and CSF NfL levels across all patient groups, nor was there any correlation with clinical data within the ALS patient group.

This study represents the first attempt to use peripherin as a fluid biomarker and to compare its levels with those of a more established biomarker such as NfL. The substantial neurodegeneration evident in MND patients likely enabled the use of ELISA, a technique characterized by a higher detection threshold compared to other methods like Simoa, for its quantification.

### 5.2 Traumatic Axonal Injury

Another condition in which peripherin has been tested as a biomarker is TAI. In an initial study, Liang and colleagues analysed the differential expression of cytoskeletal proteins in TAI animal models using proteomics and identified peripherin as a potential new biomarker for TAI, as it was significantly upregulated during DAI, with increased expression in axons after injury (Liang et al. 2019).

In a subsequent study, the same group analysed both animal models of TBI and post‐mortem human tissues obtained from subjects who had experienced severe TBI, utilizing immunohistochemistry, ELISA, and morphological analysis to assess peripherin levels and distribution following a severe impact (Fang et al. 2023).

The authors demonstrated that peripherin‐positive regions were widely distributed along the axonal tracts throughout the brain, and axonal lesions with peripherin inclusion were observed after TBI. Additionally, peripherin was found to be significantly increased in both cerebrospinal fluid and plasma in the early post‐TBI phase in animal models. Furthermore, colocalization analysis based on microscopy revealed that peripherin serves as an immunohistological biomarker in the neuropathological diagnosis of TAI. Finally, the part of the study evaluating brain samples from TBI patients, conducted with immunohistochemistry of peripherin, neurofilament heavy chain, APP, and NfL on human brain tissues, further confirmed peripherin as an immunohistological biomarker applicable in clinical practice (Fang et al. 2023).

### 5.3 Peripherin as a Possible Biomarker in Guillain Barrè Syndrome

Perhaps the most significant work published on the use of peripherin as a fluid biomarker dates to 2023. Keddie and collaborators began by exploring the distribution of peripherin in different rat tissues, demonstrating that, while NfL is present in the brain, spinal cord, and peripheral nervous system, the distribution of peripherin is restricted to the spinal cord (specifically in the anterior horn cells, motor neuron axon hillock, primary afferent sensory neurons, and dorsal and ventral roots) and the peripheral nervous system.

Through an analysis of the supernatants from two cell culture systems—one characterized by antibody‐mediated axonal damage and the other by antibody‐mediated myelin damage—along with a control culture system, the authors measured peripherin levels using the Simoa method. The peripherin levels in the supernatants collected 24 h after the injury were higher in the cultures characterized by axonal damage compared to those with myelin damage and the control cultures. Notably, these differences were more pronounced than those observed for NfL levels.

The most significant aspect of the study concerns the measurement of peripherin in the sera of patients and healthy controls. Four patient groups were examined, including those with Guillain‐Barré Syndrome (GBS), Chronic Inflammatory Demyelinating Polyneuropathy (CIDP), Multiple Sclerosis (MS), and dementia. The results were compared to those of a healthy control group. Notably, for most GBS patients, multiple longitudinal samples were available throughout the course of the disease, and for CIDP patients, two consecutive samples were available, allowing for the assessment of this biomarker's variation during disease progression.

With this foundation, the authors documented that the peak levels of peripherin were higher in patients with GBS compared to those with other neurological diseases and healthy controls. Peripherin levels were only minimally associated with those of NfL, and the two biomarkers exhibited different dynamics of increase and decrease in serum. It is important to note that, unlike NfL levels, serum peripherin levels do not appear to be influenced by the patient's age. This detail could significantly simplify the interpretation of serum concentrations of this biomarker across various disease conditions in the future. After correction for multiple comparisons, the authors did not find any significant associations between peripherin and NfL levels and the scores of clinical neuropathy assessment scales in the cohort of patients with GBS. Similarly, no associations were observed with neurophysiological subtypes, the presence of autonomic symptoms, cranial nerve involvement, or the degree of areflexia and sensory disturbances at the nadir of symptoms. However, the authors attribute this negative result to the low statistical power caused by the limited sample size, highlighting the need for further evaluations and confirmations. Finally, serum peripherin also appears to have potential future diagnostic value, particularly when considered in conjunction with serum NfL levels (Keddie et al. 2023a). It can be hypothesized that in the future, the combined assessment of serum (and in selected cases CSF) concentrations of neurofilament isoforms, and their ratio, may give a better hint in relation to the nervous system site of damage, distinguishing between predominant CNS or PNS damage. The calculation of the isoform ratio could be particularly relevant in all those cases in which both fluid biomarkers are increased and could therefore represent a useful diagnostic tool. The mechanisms related to peripherin, and NfL release are not fully understood yet. Several hypotheses have been proposed: peripherin release through complement‐mediated molecular membrane disruption and calpain activation (McGonigal et al. 2023), ischemic injury in the endoneurium associated with inflammatory oedema of proximal nerve trunks (Berciano 2024; Keddie et al. 2023b), or axonal nanoruptures following glial paranode rupture, leading to calcium influx and secondary axonal degeneration (Cunningham et al. 2023)).

However, several unresolved issues remain, including why some MS patients exhibit elevated serum peripherin levels, the half‐life and kinetics of peripherin in circulation, and the relationship between peripherin and NfL.

### 5.4 Hirschsprung Disease

HSCR is a congenital gastrointestinal disorder characterized by a high risk of neonatal bowel obstruction without early and easily accessible screening methods. Given the need for prompt diagnosis, a fluid biomarker could be valuable for excluding or confirming this diagnostic hypothesis. In this context, some researchers have investigated microRNA pathways in serum, identifying and proposing potential disease biomarkers as a non‐invasive screening method. Tang et al. involved 95 HSCR cases and 104 matched controls to focus on microRNA expression profiles in serum. The team used a TaqMan low‐density array and reverse transcription quantitative real‐time PCR (RT‐qPCR) assay. They identified five microRNAs (miR‐133a, miR‐218–1, miR‐92a, miR‐25, miR‐483‐5p) that were up‐regulated in HSCR, with statistically significant differences in their levels between the two groups. After conducting expression analysis on both internal and external individual samples, these microRNAs were considered indicative of the HSCR profile. The study demonstrated the diagnostic value of the five‐serum miRNA profiling system, which showed a sensitivity of 80%, specificity of 95%, positive predictive value of 94%, negative predictive value of 83%, and accuracy of 82.6% (Tang et al. 2014). Similarly, using a database comparing and reverse transcription‐quantitative PCR other three microRNAs were highlighted as potential peripheral biomarkers: hsa‐miR‐192‐5p, hsa‐miR‐200a‐3p, and hsa‐miR‐200b‐3p (Hong et al. 2022). Additionally, metabolic biomarkers were explored using chromatography‐mass spectrometry and multivariate statistical analysis based on the random forest algorithm. A total of 21 biomarkers were identified and the tryptophan metabolism being particularly crucial (Yang et al. 2023). Finally, an immunohistochemistry study targeting Neuroligin‐2 and examining its influence on GABAergic inhibitory synapses through ELISA assays on blood GABA levels demonstrated that reduced Neuroligin‐2 levels in aganglionic colon segments were associated with increased serum GABA levels, highlighting how blood GABA levels could serve as a disease marker (H. Yang et al. 2014). However, to date, there is no consensus on a fluid biomarker, and research remains in its early stages. Immunohistochemistry studies have shown the superiority of peripherin over cathepsin D, PGP 9.5, synaptophysin, chromogranin, MAP‐2, and Calretinin (the latter being the most frequently used in diagnosis) (Chisholm and Longacre 2016; Petchasuwan and Pintong 2000). These findings underscore the potential usefulness of this biomarker in immunohistochemical examinations (Holland et al. 2010; Szabolcs et al. 1996). The possibility of developing a serum‐based assay for this biomarker, currently limited to immunohistochemistry, could open new perspectives for the diagnosis of this disease.

## 6 Conclusion

Peripherin is a protein whose importance has been recognized for its structural functions within neuronal cells and the consequences arising from pathological gene variations. Although its discovery is relatively recent, many aspects of its biological functions remain unclear. Its expression in non‐neuronal cells and the protein interactions identified thus far expand its roles, making it a key player in various pathological conditions. The selective expression of peripherin in certain tissues and the alteration of its levels in biological fluids make it a promising disease biomarker, with a specific focus on acquired or inherited diseases characterized by involvement of the peripheral nervous system. This aspect is particularly important for the current or near future availability of treatments for the above‐mentioned diseases, which require biomarkers suitable for quantifying and monitoring their efficacy. The use of ultrasensitive measurement tools capable of detecting peripherin levels in serum and plasma, even at very low concentrations, could open new diagnostic and monitoring possibilities, extending its application and research to diseases where studies are currently limited to immunohistochemical analyses.

## Author Contributions

**Carlo Manco:** conceptualization, data curation, methodology, resources, writing – original draft. **Delia Righi:** supervision, visualization. **Guido Primiano:** methodology, supervision. **Angela Romano:** visualization. **Marco Luigetti:** visualization. **Luca Leonardi:** visualization. **Nicola De Stefano:** supervision, visualization. **Domenico Plantone:** conceptualization, data curation, methodology, visualization, writing – review and editing.

## Conflicts of Interest

The authors declare no conflicts of interest.

### Peer Review

The peer review history for this article is available at https://www.webofscience.com/api/gateway/wos/peer‐review/10.1111/ejn.70030.

## Data Availability

Data sharing is not applicable to this article as no new data were created or analysed in this study.
